# Bridging the Gap; Implementing the Electronic Referral System (eRS) to Mental Health Wards

**DOI:** 10.1192/bjo.2026.11490

**Published:** 2026-06-30

**Authors:** Vaishnavi Sornarajah, Tara Thornton, Deeksha Varma, Aditya Raheja, Yasmin Sara Lahrach

**Affiliations:** Central and Northwest London NHS Trust, London, United Kingdom

## Abstract

**Aims:**

Life expectancy in serious mental illness (SMI) can be reduced by 15–20 years on average. This is in part due to lack of integration between physical and mental health services.

Prior to this project, there was no formal referral system for mental health inpatients in Central Northwest London (CNWL) to seek advice or refer to physical health services. In the community, GPs use Electronic Referral System (eRS) for accessing secondary services. eRS provides an entirely paperless system for contacting other health professionals, using a secure system. This system also improves access and reduces the admin time required to make and chase appointments and advice requests. It is widely used by GPs.

The aim of our project was to introduce eRS onto all the wards within St Charles Hospital Mental Health Unit (MHU). This was intended to reduce the time between a referral being planned, and a response being received from physical health services, by 50% within a year.

**Methods:**

The initial stage was to collect a baseline measurement of the number of days it took between a plan being made for physical health input and receipt of a response.

We engaged stakeholders including Information technology services, General Practitioners and physical health colleagues to implement eRS in the MHU.

We collected data of the number of days it took from a plan for a physical health referral being made to response prior to eRS. We then re-audited the number of days it took once eRS was in place. The first cycle involved one ward, Redwood ward, for older adults. Following each cycle we scaled the project up, and introduced eRS to all wards at St Charles MHU.

**Results:**

Once eRS was integrated the number of days between a plan being made for a physical health referral and a response being received reduced on average by 94% from 53.95 days to 3.2 days on Redwood ward. The ward doctors provided feedback, saying “the system is much better and easier to follow”.

Data collected in St Charles MHU showed the number of days between a plan being made for a physical health referral and a response received reduced on average by 79% from 20 days to 4.4 days.

**Conclusion:**

eRS has shown itself to be helpful for improving access to physical health. There is now widespread awareness of the system among doctors at St Charles and eRS will improve integration between physical and mental health.

